# Treatment of Pediatric Pemphigus Foliaceus

**DOI:** 10.7759/cureus.45373

**Published:** 2023-09-16

**Authors:** Caden Carver, Mikole Kalesinskas, Ngawang Dheden, A. Razzaque Ahmed

**Affiliations:** 1 Dermatology, Midwestern University Arizona College of Osteopathic Medicine, Glendale, USA; 2 Department of Dermatology, Tufts University School of Medicine, Center for Blistering Diseases, Boston, USA; 3 Department of Dermatology, Barts and the London School of Medicine, Queen Mary University, London, GBR

**Keywords:** pediatric, systemic corticosteroids, immunosuppressive agents, clinical outcomes, intravenous immunoglobulin (ivig), rituximab (rtx), pemphigus vulgaris, pemphigus foliaceus

## Abstract

Pemphigus foliaceus (PF) is an autoimmune blistering disease limited to the superficial skin without mucosal involvement. It is clinically, histologically, and immunopathologically distinct from pemphigus vulgaris (PV). As data on pediatric PF is often merged with data on both pediatric and adult PV patients, isolating clinical outcomes in pediatric PF is not always possible. Therefore, the authors of this review analyzed clinical outcomes following therapy in pediatric PF patients only.

A search of databases resulted in 33 pediatric patients with PF. In total, 19 (57.6%) patients were treated with conventional immunosuppressive therapies (CISTs), which consisted of systemic corticosteroids and multiple immunosuppressive agents (ISAs). Further, 14 (42.4%) patients were treated with biologic agents, predominantly rituximab (RTX).

The mean age of those treated with biologics was 12.8 years (range = 0.88-18 years) compared to 8.9 years (range = 0.92-15 years) of those treated with CIST (p = 0.01). Treatment with biologics was initiated significantly longer after the diagnosis of PF when compared to patients treated with CIST (p = 0.003). RTX was used in all patients who received biologic therapy. Two (6%) patients also received intravenous immunoglobulin. When clinical outcomes were compared between CIST and biologic therapy, rates of clinical remission, partial remission, and relapse, were not statistically significantly different between groups. When RTX was used, rates of relapse and adverse events were higher in those treated with the lymphoma protocol (375 mg/m^2^ once weekly for four weeks) compared to those treated with the rheumatoid arthritis protocol (two doses of 1,000 mg two weeks apart) (p < 0.0001). The incidence of adverse events was statistically significantly higher in patients treated with CIST when compared to RTX (p = 0.003). These included both physical and psychological changes. The infection rate after treatment with RTX was 7.1%. These outcomes occurred during a follow-up of 12.5 months (range = 1-36 months) in the CIST group and 20.5 months (range = 6-67 months) in the biologic therapy group. The difference in the follow-up period was not statistically significant.

The literature suggests that biologics are superior to CIST in treating pemphigus patients. The results of this review suggest similar responses to therapy in pediatric PF patients treated with biologics compared to CIST. This may have been due to a limited duration of follow-up and a lack of detailed treatment outcomes in pediatric PF patients. The data in this review strongly suggests that specific treatment protocols need to be developed and implemented for pediatric PF patients. These patients are at a critical phase in life where PF therapy can influence or affect physical growth, hormonal changes, psychosocial development, and essential education.

## Introduction and background

Pemphigus foliaceus (PF) is an autoimmune blistering disease with superficial blisters, autoantibodies against desmoglein 1, and subcorneal deposition of IgG and C3 on direct immunofluorescence.

Pemphigus is exceedingly rare in the pediatric population and is commonly misdiagnosed [1]. The true incidence is unknown, although PF is far less common than pemphigus vulgaris (PV). Clinical features in pediatric PF appear to be similar to adult patients, with a more favorable prognosis among children [1]. Despite this, if left untreated, PF can be fatal [1]. More common concerns in pediatric patients are the deleterious effects of PF and its therapy on physical growth, psychosocial development, education, and overall quality of life [1].

Systemic corticosteroids are often considered as first-line therapy [2]. Adjunctive treatments include immunosuppressive agents (ISAs) such as azathioprine and mycophenolate mofetil. Dapsone and sulphapyridine have also been reported to treat pediatric PF [2].

Newer biologic agents, including intravenous immunoglobulin (IVIg) and rituximab (RTX), are currently used, but data is limited [1]. In the literature, PF is frequently grouped with PV. In some studies, pediatric patients are grouped with adult patients. These issues make it difficult to isolate, identify, and observe specific clinical responses for pediatric PF. The purpose of this review was to analyze clinical responses to treatment in pediatric PF patients so that future protocols may be designed.

## Review

Methodology

**Figure 1:**
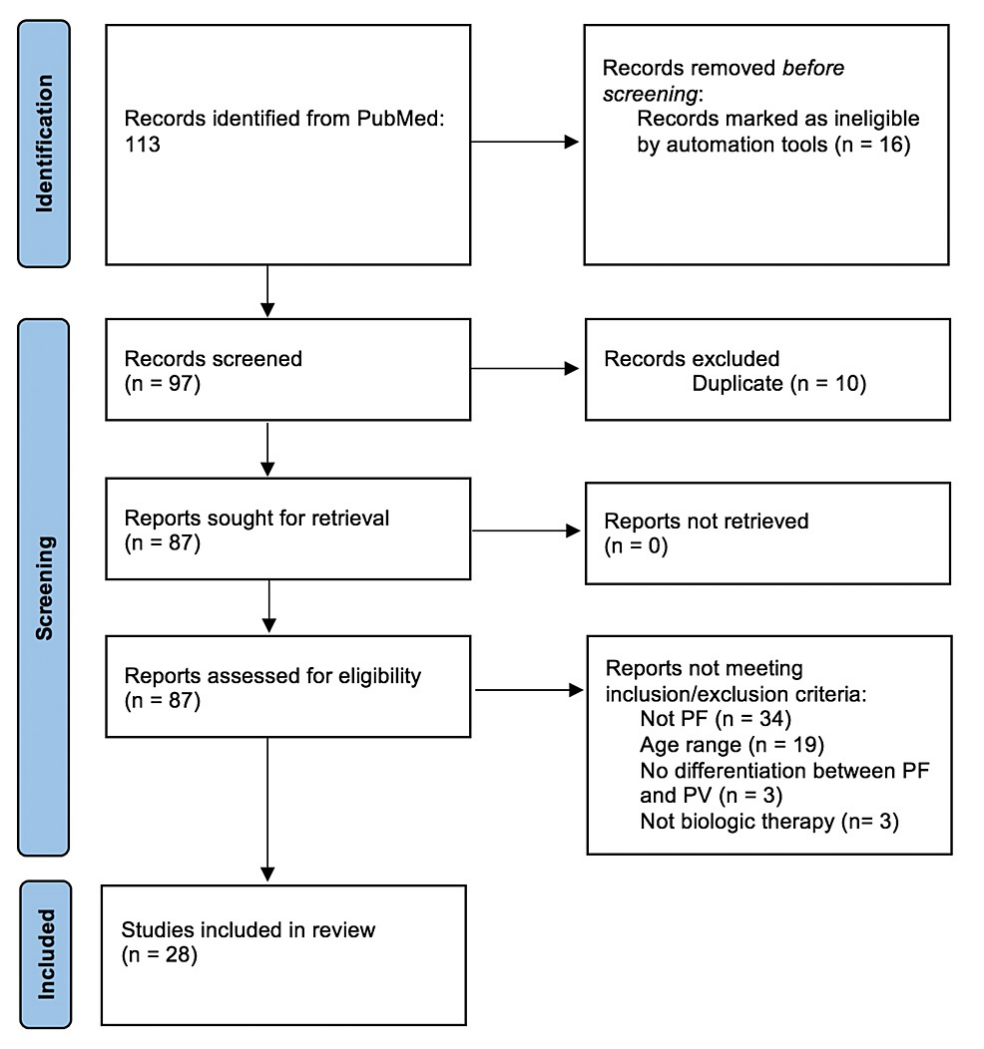
PRISMA flow diagram detailing the process of article identification and screening. PF: pemphigus foliaceus; PV: pemphigus vulgaris; PRISMA: Preferred Reporting Items for Systematic Reviews and Meta-Analyses

PubMed and Embase databases were searched using keywords pemphigus, foliaceus, adolescent, pediatric, childhood, juvenile, rituximab (RTX), intravenous immunoglobulin (IVIg), and biologic therapy. The article screening process is shown in Figure 1. Inclusion criteria were patients aged 0-18 years diagnosed with PF based on clinical, histologic, immunopathologic, and serologic criteria who were treated with conventional immunosuppressive therapies (CISTs) or biologics. Only studies published in the English language were included. Studies failing to differentiate clinical outcomes of PF from PV were excluded. Studies on endemic PF (fogo selvagem) and Tunisian PF were also excluded. A total of 28 studies were included in this review, which produced the clinical courses of 33 pediatric patients who met these criteria and were analyzed. Statistical analysis was performed using MedCalc Software, Version 22.009 [3].

Patient characteristics

A total of 33 patients [4-31] were included in this review, with a mean age of 10.6 years (range = 0.875-18 years) (Table 1). In total, 14 (42.4%) were female, and 19 (57.6%) male. The mean duration of PF at treatment initiation was 18.6 months (range = 0.25-135 months). The distribution of disease was reported in 23 patients (Table 2). Of these, in 19 (82.6%) there was involvement of the face, head, or neck. Seventeen (73.9%) had involvement of the trunk, 15 (65.2%) had involvement of the extremities, and two (8.7%) had involvement of the pubic region or perineum. Seventeen (73.9%) patients had involvement of three or more body regions of the body surface. Nineteen (57.6%) patients were treated with CIST. Fourteen (42.4%) patients were treated with one or more biologic agent/(s) in addition to CIST.

**Table 1:** Baseline characteristics of pediatric PF patients including age, sex, and disease duration at treatment initiation in those treated with CIST and RTX. CIST: conventional immunosuppressive therapy; RTX: rituximab; M: male; F: female; NA: not applicable

Regimen	Patients treated, N = 33 (%)	Mean age at treatment onset, years (range)	Sex (M/F)	Mean duration of disease at treatment onset, months (range)
CIST	19 (57.6%)	8.9 (0.92–15)	10 F/9 M	9.0 (0.25–48)
RTX	14 (42.4%)	12.8 (0.88–18)	4 F/10 M	48.7 (8–135)
P-value	NS	0.01	NA	0.003

**Table 2:** Distribution of disease for all patients, those treated with CISTs, and those treated with RTX. CIST: conventional immunosuppressive therapies; RTX: rituximab; NR: none reported

Disease distribution	All patients, N = 23 (%)	CIST, N = 19 (%)	RTX, N = 4 (%)
Face, head, or neck	19 (82.6%)	15 (78.9%)	4 (100%)
Trunk	17 (73.9%)	15 (78.9%)	2 (50%)
Extremities	15 (65.2%)	13 (68.4%)	2 (50%)
Pubic or perineal	2 (8.7%)	2 (10.5%)	NR
≥3 regions involved	17 (73.9%)	15 (78.9%)	2 (50%)

Conventional Immunosuppressive Therapy

The mean age at initiation of therapy was nine years (range = 0.92-15 years) (Table 1). Sixteen (84.2%) patients were younger than 13 years old, and three (15.8%) were aged 13-18 years. Ten (52.6%) patients were female, and nine (47.4%) male. The mean duration of disease at initiation of treatment was nine months (range = 0.25-48 months).

Biologic Therapy

The mean age in those treated with RTX was 12.8 years (range = 0.88-18 years) (Table 1). Five (35.7%) patients were less than 13 years of age, and nine (64.3%) were between 13 and 18 years. Four (28.6%) patients were female, and 10 (71.4%) were male. Indications for treatment included disease that was severe, nonresponsive to CIST, or patients with multiple relapses. In addition to CIST, RTX was used in all cases of biologic therapy, while IVIg was also used in two cases [4,25]. Five patients were treated with RTX as first-line therapy [5,28]. RTX was initiated at a mean of 48.7 months (range = 8-135 months) after the diagnosis.

Treatment regimens

Conventional Immunosuppressive Therapy

Oral prednisone or prednisolone were most commonly utilized. Doses ranged from 25 to 240 mg daily. One patient received oral betamethasone 4.5 mg daily [19]. Two patients were treated with intravenous corticosteroid pulse doses with either methylprednisolone or betamethasone of up to 500 mg [11,25]. Topical corticosteroids were reported in some cases without improvement. Therapies given in addition to corticosteroids included azathioprine, mycophenolate mofetil, dapsone, hydroxychloroquine, and sulphapyridine.

Biologic Therapy

A total of nine (64.3%) patients were treated with CIST before the addition of biologic therapy. All patients received concomitant systemic corticosteroids or ISAs with biologics. Six (42.9%) [4,5,22,25,31] were treated with RTX using the lymphoma (LP) protocol (375 mg/m^2^ once weekly for four weeks), and seven (50%) [6,28,30] were treated with the rheumatoid arthritis (RA) protocol (two doses of 1,000 mg two weeks apart). One (7.1%) [7] received RTX using an unspecified protocol.

A total of two (14.3%) patients were treated with IVIg before RTX or as concomitant therapy to RTX [4,25]. Both had severe, widespread involvement and required multiple CISTs. The dosage, duration, and the total number of IVIg cycles was not clearly specified.

Clinical outcomes

Conventional Immunosuppressive Therapy

In total, 14 (73.7%) patients had complete remission (CR) (Table 3). Four (21.1%) had CR off-therapy and 10 (52.6%) had CR on-therapy. Three (15.8%) had partial remission (PR), and two (10.5%) were non-responders. The mean follow-up for this group was 12.5 months (range = 1-36 months).

**Table 3:** Clinical outcomes of patients treated with CISTs or RTX. CR: complete remission; CR off: complete remission off therapy; CR on: complete remission on therapy; PR: partial remission; NS: not statistically significant; CIST: conventional immunosuppressive therapy; RTX: rituximab

Therapy	CR, n (%)	CR off, n (%)	CR on, n (%)	PR, n (%)	No response, n (%)	Relapses, total number (%)	Adverse events, total number (%)	Follow-up, months (range)
CIST	14 (73.7%)	4 (21.1%)	10 (52.6%)	3 (15.8%)	2 (10.5%)	8 (42.1%)	11 (57.9%)	12.5 (1–36)
RTX	12 (85.7%)	4 (28.6%)	6 (42.9%)	1 (7.1%)	None reported	10 (71.4%)	1 (7.1%)	20.5 (6–67)
P-value	NS	NS	NS	NS	<0.0001	NS	0.003	NS

Biologic Therapy

All patients had clinical improvement (Table 3). Twelve (85.7%) reached CR. Four (28.6%) had CR off-therapy, and six (42.9%) had CR on-therapy. One (14.3%) patient had PR, and one (7.1%) had only clinical improvement, which did not specify CR or PR.

The RA protocol resulted in CR in six (85.8%) patients (Table 4). Two (28.6%) had CR off-therapy, and three (42.9%) had CR on-therapy. One (14.3%) patient had PR. The LP protocol resulted in CR in five (83.3%) patients. One (16.7%) had CR off-therapy, and three (50%) had CR on-therapy. One (16.7%) patient reported clinical improvement, which did not specify CR or PR. One patient had CR off-therapy following an unspecified protocol. The mean follow-up for the biologic therapy group was 20.5 months (range = 4-67 months).

**Table 4:** Clinical responses of patients treated with RTX using the RA protocol or LP protocol. RA: rheumatoid arthritis; LP: lymphoma; CR: complete remission; CR off: complete remission off therapy; CR on: complete remission on therapy; PR: partial remission; NS: not statistically significant; RTX: rituximab

RTX protocol	CR, n (%)	CR off, n (%)	CR on, n (%)	PR, n (%)	Relapse, total number (%)	Adverse events, n (%)	Follow-up duration, months (range)
RA, N = 7	6 (85.8%)	2 (28.6%)	3 (42.9%)	1 (14.3%)	2 (20%)	NR	19.6 (4–38)
LP, N = 6	5 (83.3%)	1 (16.7%)	3 (50%)	NR	7 (70%)	1 (16.7%)	21.7 (6–67)
P-value	NS	NS	NS	<0.0001	0.03	<0.0001	NS

Kianfar et al. reported two patients, aged 14 and 16 years, who were treated with RTX as first-line therapy using the LP protocol and concomitant systemic corticosteroids due to relatively severe disease [5]. These patients each had CR on-therapy, but later developed glaucoma and osteopenia from concomitant systemic corticosteroid use [5]. Both patients relapsed, and one had two relapses. Their follow-ups were 16 and 19 months [5].

Verma et al. reported three patients who were given RTX as first-line therapy [28]. All three patients were treated with the RA protocol and concomitant CIST, which resulted in CR on-therapy with no relapse. Follow-ups occurred over 24, 36, and 38 months. No adverse events were reported.

Connelly et al. reported a 21-month-old female, who was refractory to a combination of IVIg, systemic corticosteroids, and ISAs [5]. She was then treated with 12 doses of RTX, with associated B-cell depletion leading to CR on systemic corticosteroid therapy. The patient had no relapses during a follow-up of six months.

Teo et al. reported a nine-year-old male with PF who failed to respond to initial treatment with high-dose systemic corticosteroids and ISAs [25]. A combination of monthly IVIg (2 g/kg) and systemic corticosteroids also failed to induce remission. RTX was added using the LP protocol, which resulted in CR for 1.5 years. The patient had multiple relapses, requiring four additional cycles of IVIg, RTX, and systemic corticosteroids. This was followed by RTX as maintenance therapy with 500 mg RTX every six months. The patient had no adverse events during his treatment course, with a total follow-up of approximately 60 months after the first RTX infusion.

Relapse

Conventional Immunosuppressive Therapy

The relapse rate was 42.1% (Table 3). Seven (87.5%) patients were less than 12 years of age and one (12.5%) was older than 12. Relapse occurred at a mean of 8.5 months (range = 1-19 months) after initial therapy.

Biologic Therapy

The relapse rate was 71.4% (Table 3). One (10%) patient had four relapses [25], and one (10%) patient had two [5]. Four (80%) relapsing patients were older than 12 years of age, while one (16.7%) was less than 12 years of age. The time course was only reported in one patient, who relapsed 19 months after RTX [25].

Adverse events

Conventional Immunosuppressive Therapy

A total of 11 adverse events were reported (Table 5). Six (31.6%) [9,15-18,21] patients developed cushingoid features and weight gain. Other adverse effects were Cushing syndrome [12], growth retardation [20], anemia [21], mood changes [24], and gastrointestinal disturbances [24] (Table 5).

**Table 5:** Adverse effects from treatment with CIST or RTX. NR: not reported; CIST: conventional immunosuppressive therapy; RTX: rituximab

Adverse effects following treatment	CIST, N = 19 (%)	RTX, N = 14 (%)
Cushingoid appearance/central adiposity	6 (31.6%)	NR
Cushing syndrome	1 (5.3%)	NR
Growth retardation	1 (5.3%)	NR
Anemia	1 (5.3%)	NR
Irritability	1 (5.3%)	NR
Gastrointestinal disturbance	1 (5.3%)	NR
Infection	NR	1 (7.1%)

Biologic Therapy

One (7.1%) patient had an infection [4] (Table 5). The patient developed bacteremia after a central port infection, requiring hospitalization and intravenous antibiotics. Notably, this patient was less than one year old and had received 12 RTX doses, IVIg, and systemic corticosteroids.

Discussion

PF is rare in the general population, especially in pediatric patients. Clinical studies and reviews frequently combine PF patients with PV patients. PF is clinically, histologically, and immunopathologically distinct from PV. Pediatric PF needs to be differentiated from adult PF because of special circumstances in pediatric patients that warrant attention and specific approaches to treatment and management.

Treating autoimmune bullous diseases in the pediatric population has unique challenges compared to the adult population. Hence, choosing an effective regimen for these patients is also difficult. Although systemic corticosteroids are commonly used as first-line therapy, pediatric patients are especially vulnerable to their adverse effects. Biologic therapies are increasingly used and may offer alternatives to circumvent the use of high-dose systemic corticosteroids.

In clinical practice, systemic corticosteroid use helps in clinical improvement, but disease recurrence occurs as doses are lowered. Therefore, the dose must be increased again. This cycle repeats itself many times. The addition of ISAs, such as azathioprine, mycophenolate mofetil, or methotrexate may reduce recurrence, although the disease may also flare as ISAs are decreased or discontinued. This pattern of clinical course was not reported in patients in this review, with many potential explanations. Some of these are that the limited follow-up did not allow for evaluation of the entire clinical course, including relapse rate. As pediatric pemphigus affects a different age group, it may vary in clinical course compared to adult pemphigus.

Data on 33 pediatric PF patients was analyzed in this review. These patients were divided into two groups. Nineteen (57.6%) patients were treated with only conventional ISAs (CIST). Fourteen (42.4%) patients were treated with RTX, who had previously received CIST. Only two (6%) patients received intravenous IVIg during their treatment course [4,25]. Biologics were utilized largely following the failure of CIST, although five patients were treated with RTX as first-line therapy [5,28].

Following treatment with CIST, 89.5% of patients had clinical improvement. Fourteen (73.7%) had CR, three (15.8%) had PR, and two (11.5%) failed to respond. Eight (42.1%) relapses and 11 (57.9%) adverse events occurred over a mean follow-up of 12.5 months (range = 1-36 months).

When the duration between the establishment of the diagnosis of PF and the onset of systemic therapy was studied, the duration of those treated with RTX compared to those treated with CIST was longer. This difference was statistically significant (p = 0.003). When the mean age at initiation of treatment was studied, patients who received RTX were older than those treated with CIST only. This difference was statistically significant (p = 0.01). All patients in the RTX group had clinical improvement. Twelve (85.7%) of them had CR, and two (14.3%) of them had PR. When the rates of CR, CR off-therapy, CR on-therapy, and PR were compared in the RTX group to the CIST group, no statistically significant differences were observed. Rates of CR and PR were similar between patients treated with the LP protocol compared to the RA protocol. Ten (71.4%) relapses were reported during a mean follow-up duration of 20.5 months (range = 6-67 months) in the RTX group. One (7.1%) patient had a serious bacterial infection, requiring hospitalization and intravenous antibiotics. Both relapses and adverse events occurred only in the patients treated with the LP protocol. Significantly more adverse events were reported in the CIST group compared to the RTX group (p = 0.003). No infections were reported in the CIST group. However, this could be entirely due to the fact that the follow-up period in this group was extremely limited.

Side effects were reported in 39.4% of all PF patients. However, 84.6% of these side effects were reported in patients in the CIST group. These included cushingoid features, Cushing syndrome, growth retardation, anemia, irritability, and gastrointestinal upset. These adverse events are especially concerning in pediatric patients, with possible long-term physical effects, psychological damage, and interruptions to their education secondary to them. Nonetheless, it should be noted that previous studies did not address these issues. While fewer adverse effects were reported in the RTX group, B-cell depletion was associated with serious infection in one case.

A review of pediatric PV patients reported that 71.7% had CR when treated with RTX [32]. Most patients were refractory to CIST or had side effects from its use as previous therapy, yet were treated with CIST as concomitant therapy with RTX. The relapse rate was 26.1%, and the infection rate was 10.9%. The mortality rate was 2.2%. These patients were followed for a mean of 30 months (range = 5-103 months).

A review of adults with PF refractory to CIST reported CR in 50% after RTX and CIST. These patients were also treated with concomitant CIST. The relapse rate was not reported. The infection rate was 42%. These patients were followed for a mean of 19.5 months [33].

There may be many reasons for the infrequent use of IVIg in treating pediatric PF patients. One of these reasons could be concerns about the cost of IVIg. A study comparing the cost of IVIg to CIST in treating autoimmune mucocutaneous blistering diseases reported that the total cost of IVIg was significantly less than that of CIST [34]. This analysis accounted for the cost of treating relapses and adverse events in addition to the cost of the drug itself [34]. If the same paradigm is used to compare IVIg to RTX, the longitudinal costs of treating relapse and infection for each should be considered. When IVIg has been used to treat pemphigus in adults, either as monotherapy or in combination with RTX, it resulted in sustained clinical remission with no infections or adverse events, while eliminating the use of CIST [35].

The major limitation of this study was the small sample size. This limitation was due to the fact that PF is rare in pediatric patients. Another limitation was the lack of long-term follow-up. Time courses for clinical response and relapse were not frequently reported, limiting the analysis of these aspects. Given the recalcitrant course of PF patients, it was challenging to determine the precise role that each agent had in clinical outcomes. Because RTX is a B-cell-depleting agent, the lack of studies on B-cell levels was particularly concerning. The lack of autoantibody titers also made it difficult to determine whether CIST or RTX had a better impact on the reduction of autoantibody titers and possibly elimination. Furthermore, it would have been very useful if relapses were correlated with B-cell repopulation and a rise in autoantibody titers. Such information would guide specific dosing of biological agents and the timing of their use. Additionally, many studies did not report the dose of systemic corticosteroids as mg/kg/day. A significant limitation of the data is the lack of studies on the impact of PF on psychosocial development and possibly anxiety, depression, and isolation that patients may suffer due to visible cutaneous disease.

## Conclusions

It is necessary for the clinician who encounters blisters in a pediatric patient to keep PF as their differential diagnosis. The first step should be to rule out infection or gluten intolerance. If blisters fail to respond to initial treatments, an autoimmune workup should be considered. As PF is confined to the skin, the role of topical therapies should be heavily emphasized. Pediatric patients may be especially susceptible to exacerbations of PF, resulting from trauma and solar exposure. Medical and non-medical therapies should both aim to improve disease control and quality of life. In a disease that fails to respond to conservative management and topical therapy, the clinician must be informed in considering additional options, depending on the age of the patient as well as disease severity. While systemic corticosteroids may have some role initially in controlling lesions, prolonged courses and high doses may be especially harmful in the pediatric population. ISAs are valid adjuvants. Caution is needed to prevent prolonged immune suppression and opportunistic infection. The use of RTX should be made with deliberation and concern for its adverse events, primarily infection. Although data on its use in pediatric PF is somewhat limited, IVIg may be beneficial for immunoprophylaxis. IVIg also has anti-inflammatory properties and can restore immune balance while producing sustained clinical remission.

The data analysis in this review would indicate that pediatric PF patients were treated with protocols similar to adult PV. This outcome may have been due to the fact that many studies included PF patients with PV and that in some studies, pediatric patients were included with adult pemphigus patients. Therefore, this review clearly highlights that the treatment of pediatric PF needs to be revisited and redefined. A consensus-building conference that includes pediatric dermatologists, psychiatrists, immunologists, and clinical pharmacologists is needed to produce specific protocols for treating pediatric PF. These protocols should be based on the age and severity of disease for each patient. Such treatments should produce rapid clinical control of disease while minimizing adverse events. Specifically, these therapies should not disturb or derange the physical, psychological, emotional, and social growth and development of pediatric patients. Treatments must not only consider clinical improvement and recovery but also emphasize quality of life with equal importance.
